# Supplementary material for: Upper airway gene expression reveals a more robust innate and adaptive immune response to SARS-CoV-2 in children compared with older adults
Source: Res Sq. 2021 Aug 26:rs.3.rs-784784. Preprint. [Version 1] doi: 10.21203/rs.3.rs-784784/v1 (PMC8404906; doi:10.21203/rs.3.rs-784784/v1)
Supplement: Supplement 6 — Figure 3–figure supplement 1: Additional cell-types. Additional cell types included in the cell-type proportions analysis. [file NIHPPrs784784v1-supplement-6.pdf]

Supplemental Tables

Supplemental Table 1A: Adult vs Pediatric cohort characteristics.

	Adults (All)	Children (All)	*p* value	Adults (SARS-CoV-2)	Children (SARS-CoV-2)	*p* value
Total Enrolled (n)	154	83		45	38	
Age, years (median, range, IQR)	62 (40-89, 47-71)	4 (<1-19, 2-12)		57 (40-82, 44-64)	5 (<1-19, 2-11)	
Female gender	78 (51%)	42 (51%)	0.93	25 (56%)	16 (42%)	0.27
**Clinical Encounter Type**						
Inpatient	42 (27%)	31 (37%)		4 (9%)	1 (3%)	
Intensive Care Unit	16 (10%)	0 (0%)		2 (4%)	0 (0%)	
Emergency Department	24 (16%)	19 (23%)		3 (7%)	6 (16%)	
Outpatient	55 (36%)	31 (37%)		24 (53%)	30 (79%)	
Unknown	17 (11%)	2 (2%)	0.59	12 (27%)	1 (3%)	0.23
**Race**						
White or Caucasian	67 (44%)	34 (41%)		6 (13%)	13 (34%)	
Asian	25 (16%)	6 (7%)		6 (13%)	1 (3%)	
Black or African American	14 (9%)	4 (5%)		1 (2%)	2 (5%)	
Native Hawaiian or Pacific Islander	1 (1%)	2 (2%)		1 (2%)	0 (0%)	
American Indian or Alaska Native	0 (0%)	1 (1%)		0 (0%)	0 (0%)	
Other	27 (18%)	29 (35%)		17 (38%)	17 (45%)	
Unknown	20 (13%)	7 (8%)	**0.013**	14 (31%)	5 (13%)	0.10
**Ethnicity**						
Not Hispanic or Latino	109 (71%)	43 (52%)		16 (36%)	10 (26%)	
Hispanic or Latino	23 (15%)	34 (41%)		14 (31%)	23 (61%)	
Unknown	22 (14%)	6 (7%)	**<0.001**	15 (33%)	5 (13%)	0.06

Values are n (%) unless otherwise indicated.
*Race categories with <1 patient in both groups were excluded from analyses.
Unknown values were excluded from analyses.

Supplemental Table 1B: Adult cohort clinical and demographic characteristics.

	Cohort Overall	SARS-CoV-2	Other Virus	No Virus	*p* value
Total Enrolled (n)	154	45	28	81	
Age, years (median, range, IQR)	62 (40-89, 47-71)	57 (40-82, 44-64)	61.5 (40-89, 45-73)	62 (41-89, 52-72)	0.75
Female gender	78 (51%)	25 (56%)	10 (36%)	43 (53%)	0.21
**Clinical Encounter Type**					
Inpatient	42 (27%)	4 (9%)	9 (32%)	29 (36%)	
Intensive Care Unit	16 (10%)	2 (4%)	5 (18%)	9 (11%)	
Emergency Department	24 (16%)	3 (7%)	6 (21%)	15 (19%)	
Outpatient	55 (36%)	24 (53%)	8 (29%)	23 (28%)	
Unknown	17 (11%)	12 (27%)	0 (0%)	5 (6%)	**0.003**
**Race**					
White or Caucasian	67 (44%)	6 (13%)	19 (68%)	42 (52%)	
Asian	25 (16%)	6 (13%)	5 (18%)	14 (17%)	
Black or African American	14 (9%)	1 (2%)	1 (4%)	12 (15%)	
Native Hawaiian or Pacific Islander	1 (1%)	1 (2%)	0 (0%)	0 (0%)	
American Indian or Alaska Native	0 (0%)	0 (0%)	0 (0%)	0 (0%)	
Other	27 (18%)	17 (38%)	3 (11%)	7 (9%)	
Unknown	20 (13%)	14 (31%)	0 (0%)	6 (7%)	**<0.001***
**Ethnicity**					
Not Hispanic or Latino	109 (71%)	16 (36%)	26 (93%)	67 (83%)	
Hispanic or Latino	23 (15%)	14 (31%)	1 (4%)	8 (10%)	
Unknown	22 (14%)	15 (33%)	1 (4%)	6 (7%)	**<0.001**

Values are n (%) unless otherwise indicated.
*patients with Native Hawaiian or Pacific Islander race; and patients with American Indian or Alaska Native race were excluded from statistical analysis due to insufficient numbers.
Unknown values were excluded from analyses.
Age was analyzed as one-way ANOVA and all categorial variables using chi-squared tests.

Supplemental Table 1C: Pediatric cohort clinical and demographic characteristics.

	Cohort Overall	SARS-CoV-2	Other Virus	No Virus	*p* value
Total Enrolled (n)	83	38	11	34	
Age, years (median, range, IQR)	4 (<1-19, 2-12)	5 (<1-19, 2-11)	3 (<1-14, 2-11)	4 (<1-16, 2-12)	0.97
Female gender	42 (51%)	16 (42%)	7 (64%)	19 (56%)	0.38
**Clinical Encounter Type**					
Inpatient	31 (37%)	1 (3%)	2 (18%)	28 (82%)	
Intensive Care Unit	0 (0%	0 (0%)	0 (0%)	0 (0%)	
Emergency Department	19 (23%)	6 (16%)	8 (73%)	5 (15%)	
Outpatient	31 (37%)	30 (79%)	0 (0%)	1 (3%)	
Unknown	2 (2%)	1 (3%)	1 (9%)	0 (0%)	**<0.001**
**Race**					
White or Caucasian	34 (41%)	13 (34%)	4 (36%)	17 (50%)	
Asian	6 (7%)	1 (3%)	1 (9%)	4 (12%)	
Black or African American	4 (5%)	2 (5%)	2 (18%)	0 (0%)	
Native Hawaiian or Pacific Islander	2 (2%)	0 (0%)	1 (9%)	1 (3%)	
American Indian or Alaska Native	1 (1%)	0 (0%)	0 (0%)	1 (3%)	
Other	29 (35%)	17 (45%)	2 (18%)	10 (29%)	
Unknown	7 (8%)	5 (13%)	1 (9%)	1 (3%)	0.31**
**Ethnicity**					
Not Hispanic or Latino	43 (52%)	10 (26%)	7 (64%)	26 (76%)	
Hispanic or Latino	34 (41%)	23 (61%)	3 (27%)	8 (24%)	
Unknown	6 (7%)	5 (7%)	1 (9%)	0 (0%)	**<0.001**

Values are n (%) unless otherwise indicated.
*No pediatric patients were admitted to the intensive care unit; this category was excluded from statistical analyses.
**patients with Native Hawaiian or Pacific Islander race; patients with American Indian or Alaska Native race; and patients with Black or African American race were excluded from statistical analysis due to insufficient numbers.
All unknown values were excluded from analyses.
Age was analyzed as one-way ANOVA and all categorial variables using chi-squared tests.

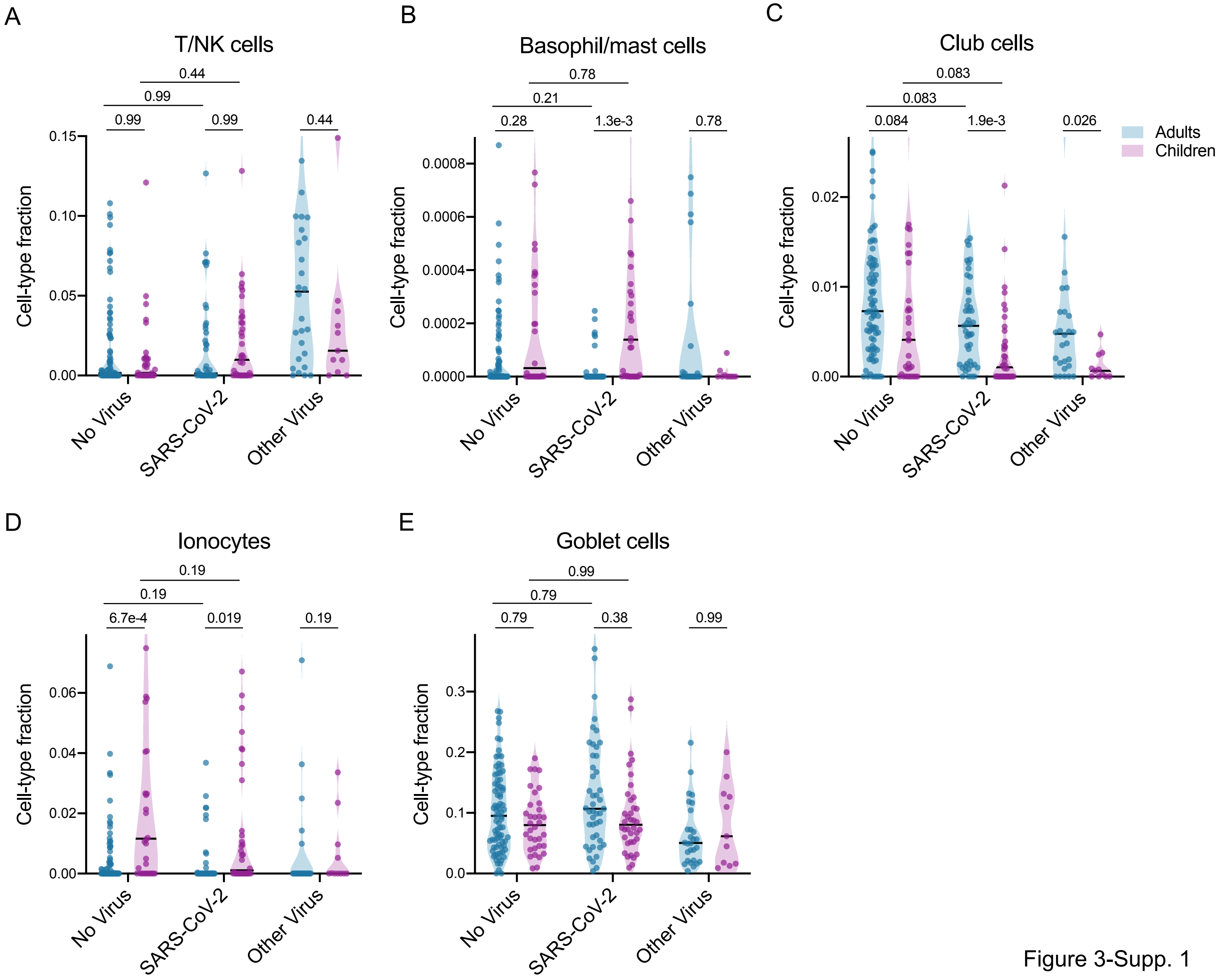

Figure 3-Supp. 1
